# Interventional strategies for ischemic stroke based on the modulation of the gut microbiota

**DOI:** 10.3389/fnins.2023.1158057

**Published:** 2023-03-02

**Authors:** Jing Wang, Xiaofeng Liu, Qun Li

**Affiliations:** Department of Gastroenterology, The 960th Hospital of the PLA, Jinan, Shandong, China

**Keywords:** ischemic stroke, gut microbiome, fecal microbiota transplantation, probiotics, traditional Chinese medication

## Abstract

The microbiota-gut-brain axis connects the brain and the gut in a bidirectional manner. The organism’s homeostasis is disrupted during an ischemic stroke (IS). Cerebral ischemia affects the intestinal flora and microbiota metabolites. Microbiome dysbiosis, on the other hand, exacerbates the severity of IS outcomes by inducing systemic inflammation. Some studies have recently provided novel insights into the pathogenesis, efficacy, prognosis, and treatment-related adverse events of the gut microbiome in IS. In this review, we discussed the view that the gut microbiome is of clinical value in personalized therapeutic regimens for IS. Based on recent non-clinical and clinical studies on stroke, we discussed new therapeutic strategies that might be developed by modulating gut bacterial flora. These strategies include dietary intervention, fecal microbiota transplantation, probiotics, antibiotics, traditional Chinese medication, and gut-derived stem cell transplantation. Although the gut microbiota-targeted intervention is optimistic, some issues need to be addressed before clinical translation. These issues include a deeper understanding of the potential underlying mechanisms, conducting larger longitudinal cohort studies on the gut microbiome and host responses with multiple layers of data, developing standardized protocols for conducting and reporting clinical analyses, and performing a clinical assessment of multiple large-scale IS cohorts. In this review, we presented certain opportunities and challenges that might be considered for developing effective strategies by manipulating the gut microbiome to improve the treatment and prevention of ischemic stroke.

## Introduction

Stroke is a devastating cerebrovascular disease characterized by high morbidity, disability, recurrence, and mortality. The data provided by the Global Burden of Disease (GBD) 2019 suggested that stroke is the second most common reason for death and the third leading reason for disability across the world. Also, the absolute number of first-ever stroke and stroke-related deaths has increased considerably over the last decade (GBD 2019 Stroke Collaborators, 2021). China has a greater burden of stroke, considering that the country has the highest prevalence of stroke in the world. Additionally, most of the years of life lost and disability-adjusted life years among Chinese adults are because of stroke (Wu et al., 2019; Ma et al., 2021; Wang Y. J. et al., 2022). Stroke can be broadly classified into ischemic and hemorrhagic stroke, with ischemic stroke (IS) contributing to more than 70% of total incidences of stroke worldwide (GBD 2016 Lifetime Risk of Stroke Collaborators et al., 2018; Tuo et al., 2022). It primarily occurs due to a cerebral arterial occlusion caused by a thrombus or embolus (Tian et al., 2019; Mistry and Dumont, 2020). Besides damaging the brain parenchyma surrounding the ischemic areas, IS also triggers complex neuropathophysiological and neuropathological events followed by neuroinflammation and immune response (Pluta et al., 2021; Zhang S. R. et al., 2021). Many recent studies have suggested that post-stroke immunosuppression and intestinal barrier damage can increase the risk of opportunistic infections after IS, which can seriously worsen the outcomes of IS (Ghelani et al., 2021). These findings indicate that effective treatment of IS and the extension of the therapeutic window are challenging, and new therapeutic strategies need to be developed.

Recanalization and neuroprotection are the main approaches for treating IS in the clinic. Performing intravenous/intra-arterial thrombolysis and mechanical thrombectomy for effective reperfusion following recanalization are necessary for a positive prognosis of IS patients (Prabhakaran et al., 2015; Wu et al., 2019). The Food and Drug Administration (FDA) has only approved intravenous recombinant tissue plasminogen activator (IV rtPA) for treating IS (National Institute of Neurological Disorders and Stroke rt-Pa Stroke Study Group, 1995). Endovascular reperfusion therapy can partially improve the overall likelihood of a good IS outcome (Prabhakaran et al., 2015; Wu et al., 2019; Saver and Adeoye, 2021). However, the overall safety and efficacy are limited by a narrow treatment window (Yeo et al., 2013) of 4.5 h from the onset of the symptoms, the challenges of cerebral ischemia-reperfusion injury (Eltzschig and Eckle, 2011; Sun et al., 2018), and the tendency of hemorrhagic transformation (Gauberti et al., 2018) during the treatment course. Therefore, many researchers are investigating novel approaches for treating IS. In the past two decades, more than 1,000 potential neuro-protectants have been found to attenuate ischemic brain injury by promoting neuronal survival, neural plasticity, neurogenesis, and synaptogenesis (Liberale et al., 2018; Shen et al., 2023). However, the studies were mainly conducted on experimental IS animal models, and only a few agents targeting these molecules could be administered in the clinic (Gauberti et al., 2018; Gan et al., 2020; Mani et al., 2023). Stem cell therapy and neural progenitor cell transplantation therapy provide a regenerative strategy for protecting neural tissue in the acute phase and the replacement of lost tissues in the sub-acute or chronic phase of IS (Wei et al., 2017; Yu et al., 2019). However, this technique has numerous challenges, including identifying suitable neural progenitors, low overall survival of the neurons, and insufficient neuronal differentiation (Wei et al., 2017; Wang S. N. et al., 2020; Mani et al., 2023). Thus, the technique needs to be further improved before clinical application.

Along with the typical neurological deficit in the acute phase (Powers, 2020), more than half of the patients with IS suffer from gastrointestinal complications, including gut motility and absorption dysfunction, intestinal bleeding, gut leakiness, and enteropathogenic sepsis (Wen and Wong, 2017). After the concept of the microbiota-gut-brain axis (MGBA) was proposed, many studies confirmed the presence of a bidirectional MGBA and the potential of microbiota-directed interventions to improve stroke outcomes (Zhao et al., 2018). Detailed studies on the underlying mechanisms might provide a theoretical basis for developing novel interventions and therapeutic strategies for IS based on microbes (Cryan et al., 2019). With the advancement of high-throughput and “-omics” technologies, especially the integration of metagenomics and metabolomics techniques, a strong correlation was found between the gut microbiota and potential risk factors for the onset, progression of pathological changes and the prognosis and recovery of IS patients (Benakis et al., 2016; Nam, 2019; Pluta et al., 2021). Several studies have shown that the gut microbiota and their metabolites might play a dual role in IS (Peh et al., 2022). As the gut microbiome is less diverse in IS patients, modulating the composition of the gut microbiome might improve the prognosis of IS patients. On the other hand, consuming foods rich in choline and L-carnitine increases the occurrence of IS due to the generation of trimethylamine-N-oxide. Meanwhile, consuming dietary fiber improves the outcomes in IS patients due to the action of short-chain fatty acid metabolites containing butyrate and propionate, derived from gut microbes (Chen et al., 2019b; Battaglini et al., 2020; Peh et al., 2022).

Several effective strategies have been proposed for treating disorders related to gut microbiota in IS patients. The gut microbiota can be modulated using two ways: (1) By identifying keystone taxa in the gut microbiome and performing interventions; (2) By altering the composition of the intestinal microbiota by single or combined use of dietary interventions, antibiotics, probiotics, fecal microbiota transplantation (FMT), or traditional Chinese medication (TCM). Several studies have also suggested that repairing the damaged intestinal mucosal barrier by gut-derived stem cell transplantation might be a new treatment strategy, which could prevent the occurrence of endotoxemia and secondary infections. Therefore, in this review, we discussed intestinal microbiota as an intervention technique for treating IS to gain further insights into the emerging field of IS therapy.

## Dietary interventions in IS

Diet directly affects the composition of the gut microbial communities and the production of metabolites. Cellular stress caused by unhealthy diets, such as a high intake of high-fat foods, animal byproducts, and processed foods, may influence abnormal lipid metabolism and cerebral small vessel disease, which can trigger the neuroinflammatory process and, as a result, activate a neurodegenerative cascade (Nassir et al., 2021; Flaig et al., 2023). Foods high in choline and L-carnitine, such as red meat, can be metabolized by intestinal microbiota to produce trimethylamine N-oxide (TMAO), which has been shown in experimental and clinical studies to promote the occurrence of atherosclerosis and stroke (Koeth et al., 2013; Zhu et al., 2021). Reduced reverse cholesterol transport induced by TMAO *via* gut flora-related pathways is one possible mechanism (Zhu et al., 2021; Peh et al., 2022). Meanwhile, the presence of specific bacterial species in human feces has been linked to TMAO plasma concentration and diet pattern (Peh et al., 2022). TMAO may also promote platelet hyperreactivity and thrombosis by increasing Ca^2+^ release from intracellular stores during submaximal agonist stimulus-dependent platelet activation (Zhu et al., 2016). Clinical trials confirmed that plasma TMAO levels could independently predict the risk of thrombosis, including heart attack and stroke (Tang et al., 2013; Zhu et al., 2016; Wang M. et al., 2022). Furthermore, TMAO-mediated pathogenesis is associated with the activation of multiple inflammatory signaling pathways, which may result in oxidative stress, mitochondrial dysfunction, neuronal aging, synaptic compromise, and cognitive impairment (Praveenraj et al., 2022).

Consumption of dietary fiber and polyphenols, on the other hand, may improve stroke outcomes *via* gut flora-associated SCFAs such as butyrate and propionate (Fraga et al., 2019; Peh et al., 2022). Long-term consumption of short-term fermented soybeans (chungkookjang) containing specific Bacillus species in animal models of stroke could influence host metabolism, particularly inflammation and insulin resistance, through regulation of gut microbiota composition (increase in *Lactobacillus*, *Bacillus*, and *Akkermansia*) and metabolites (increase in propionate and butyrate), and further prevent neuronal cell death and memory dysfunction from the artery occlusion (Zhang T. et al., 2021). Nonetheless, the underlying mechanisms are unknown. In a recent study, sodium butyrate was shown to reduce neuronal apoptosis by activating PI3K/Akt *via* the G protein-coupled receptor GPR41/Gβγ in a rat model (Zhou et al., 2021).

Collectively, dietary intervention may be an appealing and valuable way to influence the course of IS.

### Dietary patterns in the prevention of IS

Many studies have shown the importance of overall dietary patterns in the prevention and reduction of the occurrence of IS. Diet quality and unbalanced nutrition are risk factors that strongly increase the chances of the incidence of a first-ever stroke (O’Donnell et al., 2016), as well as the relapse of stroke and other vascular events (Amarenco et al., 2016; Yusuf et al., 2020). A study found that compared to not consuming vegetables, consuming 306–372 g of vegetables can reduce the risk of IS by 23.2%. The results indicated that vegetable consumption could effectively protect people from IS (Stanaway et al., 2022). Additionally, long-term dietary habits and the intensity of systemic inflammation were found to be strongly correlated, suggesting that the diet can modulate carotid plaque vulnerability in IS patients (Peng et al., 2020). In that study, Peng et al. (2020) calculated the dietary inflammatory index (DII) of 32 food components with a detailed questionnaire on food frequency. They found that IS patients who consumed foods with lower anti-inflammatory properties, including fruits, vegetables, and nuts, had a higher DII score and were vulnerable to plaques.

In the IS population, evaluating whole dietary patterns is more promising than evaluating individual nutrients or food components. The EAT-Lancet Commission proposed an integrated framework related to a health-reference diet based on a sustainable food system to achieve better overall health outcomes and to conform to food culture in most parts of the world. Individualizing energy intake based on body size, body composition, and physical activity levels was recommended (English et al., 2021; Wu and Anderson, 2021). English et al. (2021) evaluated the information related to the dietary patterns affecting primary and secondary stroke prevention, and they recommended that the most effective dietary strategies include following the Mediterranean diet, low sodium intake, and intake of folic acid supplements in regions with low folate. To address the complexities and the insufficient evidence directly relevant to clinical implications, well-designed randomized controlled trials need to be conducted based on appropriate dietary interventions, especially for people who have suffered a stroke.

The effects of drinks have also been investigated. According to a 16-year follow-up study, drinking water with a high concentration of calcium and magnesium (magnesium ≥10 mg/L or calcium ≥50 mg/L) is related to a lower risk of IS. The study also showed that drinking water enriched with calcium and magnesium, especially magnesium, can significantly reduce the risk of IS in postmenopausal women (Helte et al., 2022). Coffee and tea are extremely popular beverages globally and possess health benefits. A large prospective cohort study conducted with 365,682 participants from the UK Biobank showed that drinking 2–3 cups of coffee or tea per day decreased the risk of stroke by 32% during the median follow-up of 11.4 years for new onset IS. People who consumed both coffee and tea, particularly up to 3–6 cups daily, had the lowest risk of IS and vascular dementia after a stroke (Zhang Y. et al., 2021).

### Dietary alteration accompanied by shifts in the intestinal metabiome

The gut microbiota encodes many carbohydrate-active enzymes. Dietary fiber and carbohydrates in the diet can be fermented to produce short-chain fatty acids (SCFAs) through these enzyme systems. Many studies have shown that SCFAs can regulate immune responses, maintain gut barrier integrity, suppress the activity of histone deacetylases, and block the cascade of inflammatory reactions (Kasahara and Rey, 2019). Sodium butyrate (NaB; an SCFA) is a histone deacetylase inhibitor generated by butyrate-producing bacteria (BPB). NaB can cross the blood-brain barrier (BBB) and lower oxidative stress in the brain, subsequently increasing the expression level of the neuroprotectant IGF-1 in peripheral tissues (Park and Sohrabji, 2016), reducing the expression of proinflammatory cytokines in the serum (Wang H. et al., 2022), and ultimately effectively decreasing brain injury after a stroke. Therefore, it can aid in neurological recovery and treat cognitive impairment following a stroke (Wang H. et al., 2022). Furthermore, when a moderate amount of fiber, butyrate, or probiotic-producing butyrate is added to the diet, the leaky gut can be repaired in IS patients (Boivin et al., 2016), and the consolidated integrity of the epithelial barrier can provide neuroprotection during stroke recovery. Also, consuming fermented dairy foods, including cheese and yogurt, which contain beneficial probiotics (Aryana and Olson, 2017), can help in the prevention and treatment of IS (Zhang K. et al., 2020) by improving the overall intestinal microbiota (Carr et al., 2021) after the living microorganisms reach the intestine.

Additionally, moderate restriction in dietary proteins and energy can provide neuroprotection by modulating the gut microbiota. In the mouse model of middle cerebral artery occlusion, the effects of a moderately low protein diet on decreasing the cerebral infarction volume and restoring neuroplasticity were associated with higher antioxidant reactions, lower neuroinflammation, and rebalanced commensal gut microbiota in the post-acute phase (Silva de Carvalho et al., 2022). Calorie restriction was also reported to enhance post-stroke rehabilitation, which might correlate with the dramatically altered composition of the gut microbiota and its metabolism, in which *Bifidobacterium* was enriched (Huang et al., 2021). These findings might provide novel strategies for stroke rehabilitation in the clinic based on diet control and gut microbiota.

### Enteral nutrition (EN) in IS

The stress status during the acute phase of stroke is characterized by high decomposition and high metabolism. It can trigger hyperglycemia, acidosis, hypoproteinemia, and negative nitrogen balance, leading to serious malnutrition, weakening the immune system, and increasing complications (Xu C. Y. et al., 2021). Many studies have proposed the concepts of immune and microecological nutrition, and the latter’s role was found to be especially important. For stroke patients, early EN combined with probiotics can help in improving the nutritional status, reconstructing the gut microbiota, stabilizing intestinal barrier function, improving immune tolerance, and decreasing the complications of infection and nutritional diarrhea, thus, facilitating a more effective therapeutic intervention (Xu and Shao, 2015; Mao et al., 2022). Furthermore, systematic reviews and meta-analyses of randomized controlled trials have confirmed the efficacy of EN in IS patients (Chen et al., 2022; Savigamin et al., 2022). On the other hand, additional high-quality and well-designed randomized controlled trials are required to provide more reasonable theoretical guidance for clinical practice (Chen et al., 2022).

## Administration of antibiotics in IS

Many studies have investigated the application of antibiotics to prevent post-stroke infections and improve stroke outcomes. According to some studies, post-stroke immunodepression and stress can disrupt the intestinal epithelial barrier and facilitate the spread of commensal bacteria from the host gut microbiota, causing systemic infections (Kumar et al., 2010). Infections, particularly pneumonia, commonly occur after a stroke and might contribute to neurological deficits and an increase in the mortality rate (Faura et al., 2021). Therefore, antibiotics are currently used in clinical practice to prevent infections following stroke; a common approach involves the use of broad-spectrum antimicrobial agents or combinations (Westendorp et al., 2015, 2018). Antibiotics are often administered for the early prevention and control of IS, and for patients with severe IS, broad-spectrum antibiotics are usually administered for 1 week (Meisel and Smith, 2015). However, the safety and efficacy of prophylactic antibiotics used for treating IS remain unclear. Besides their role in antimicrobial prophylaxis, antibiotic intervention can also change the composition of the intestinal microbiota and disturb the homeostasis of the microbiota for several months or even years (Langdon et al., 2016; Rizzatti et al., 2018). This might, in turn, increase the risk of infection, particularly pneumonia, as the disturbance or even eradication of the commensal bacterial communities might lead to the production of bacterial fragments, which can act as toxins and co-stimulants (Winek et al., 2016). Several studies have evaluated the necessity of administering prophylactic antibiotics to IS patients in intensive care units. Early prophylactic antibiotic treatment with ceftriaxone (cephalosporin), levofloxacin (fluoroquinolone), penicillin, and minocycline (tetracycline), most of which were prescribed within 24 h, could not reduce the occurrence of post-stroke pneumonia or the mortality rate in a longer follow-up, despite decreasing the incidence of urinary tract infections and other post-stroke complications (Zheng et al., 2017; Rashid et al., 2020; Wang Q. et al., 2022).

However, preventive antibiotic therapy at the onset of a stroke is still important. For example, the prophylactic use of antibiotics is highly efficient in specific subgroups of IS patients (Vermeij et al., 2018). Liu C. et al. (2022) showed that broad-spectrum antibiotics could decrease systemic and brain cytokine levels, decrease infarct size and perilesional cortex apoptosis, improve long-term behavioral recovery, and strongly affect the gut microbiota in rats after cerebral ischemia. Their study showed that antibiotic prophylaxis has neurorestorative benefits after IS. Their findings indicated that oral administration of non-absorbable antibiotics might strongly affect stroke pathophysiology by altering commensal gut bacteria. Benakis et al. (2020) also showed that a cocktail of antibiotics significantly decreased the infarct volume of IS mice in the acute phase. In contrast, the neuroprotective effect was abolished with the re-colonization of a wild-type gut microbiota in the model mice. They also discovered that antibiotic treatment with ampicillin or vancomycin as monotherapy, rather than neomycin, was sufficient for reducing infarct volume and improving sensory and motor function 3 days after the stroke. Furthermore, specific microbial populations, particularly Bacteroidetes S24.7, and microbial metabolites primarily containing aromatic amino acids, exerted this neuroprotective effect. These findings highlighted the preventive effects on the short-term and long-term outcomes of IS patients due to the targeted modification of the microbiome related to specific microbial enzymatic pathways following the administration of specific antibiotics.

However, further studies are needed to determine whether the administration of antibiotics can improve the outcomes of IS patients and whether antibiotics affect post-stroke infections through the intestinal flora. Also, as non-infectious inflammation comprises a significant portion of stroke-associated pneumonia due to the risk factors of dysphagia and stroke-induced immunodepression (Eltringham et al., 2020), combination therapy using antibiotics and targeted immunomodulatory agents might more effectively improve the prognosis of IS patients (Meisel and Meisel, 2011; Meisel and Smith, 2015).

## Probiotics and prebiotics in IS

According to the World Health Organization (WHO), probiotics are live microbial food supplements or components of bacteria that are beneficial to humans when administered in adequate amounts (Hill et al., 2014). Several recent studies have shown the beneficial effects of specific probiotic strains or a mixture of strains at particular life stages or disease stages. Some studies investigated the mechanism of action of probiotics in IS to elucidate how probiotics strengthen the gut epithelial barrier function, inhibit pathogen adhesion to the intestinal wall by adhering to the intestinal mucosa, suppress bacterial translocation, produce bioactive compounds, including bacteriocins, organic acids, vitamins, and neurotransmitters, reduce certain biomarkers of oxidative stress and inflammatory cytokines, produce anti-inflammatory compounds to modulate the immune system, and upregulate the expression of opioid and cannabinoid receptors in intestinal epithelial cells; thus, activating calcium-dependent potassium channels in intestinal sensory neurons (Sánchez et al., 2017; Martínez-Guardado et al., 2022). Additionally, SCFAs produced by probiotics can counteract neuroinflammation after IS (Sadler et al., 2020; Zhang W. et al., 2022) and help in repairing cognitive dysfunction and brain injury. Probiotics can also improve the negative emotions of IS patients, including anxiety and depression, 3 months after stroke (Bailey and Cryan, 2017; Liu et al., 2020). Probiotic treatment not only alters the microenvironment to limit pathological progress but also plays a complementary role by promoting the pharmaceutical management of calcium-channel blockers and statins (Liu W. et al., 2022). Combinatorial therapy with regenerative medicine, such as stem cell therapy, has also been found by some researchers to increase the level of the neurotrophic factor brain-derived neurotrophic factor (BDNF) through symbiotic treatment to enhance neurogenesis and post-stroke cognitive function. Therefore, this treatment strategy is promising and warrants further investigation (Romo-Araiza et al., 2018; Xu H. et al., 2021).

*Lactobacillus* and *Bifidobacterium* are probiotics that can hinder the overgrowth of opportunistic pathogens and the invasion of foreign pathogens, and thus, help in maintaining the intestinal microecological balance, lowering the apoptosis of intestinal epithelial cells due to pathogens, protecting the intestinal mucosal barrier, and improving the intestinal and systemic immune functions (Chen et al., 2022). Studies on rodent models have shown the beneficial effects of probiotic strains such as *Bacillus licheniformis* (Li Y. et al., 2021), *Lactobacillus* (Wanchao et al., 2018), and *Clostridium butyricum* (Sun et al., 2016) on stroke. The beneficial effects of prebiotics on IS have also been studied extensively (Hill et al., 2014; Gibson et al., 2017). Lactulose is an important prebiotic, which can elevate the levels of SCFAs in the intestine and serum (Bothe et al., 2017; Chen X. et al., 2020), aggravate post-stroke inflammation, and improve the functional prognosis of stroke (Yuan et al., 2021). Some studies have also shown that intragastric administration of indole-3-propionic acid (IPA) to mice with middle cerebral artery occlusion (MCAO) can restore the alterations in the structure of the gut microbiome with elevated probiotics and reduce the number of harmful bacteria, repair the integrity of the intestinal barrier, inhibit A1 reactive astrogliosis by regulating the activities of regulatory T cells (Tregs)/Th17 cells in gut-associated lymphoid tissue, and thus, efficiently alleviate the effects of neuritic impairment and brain infarction (Xie Y. et al., 2022). Prebiotics like functional barley can increase the number of butyrate-producing bacteria and promote the production of intestinal butyrate (Akagawa et al., 2021). Therefore, to better apply the synergistic and beneficial effects of probiotics and prebiotics on therapy, “synbiotics,” which is a mixture of active microorganisms (probiotics) and a matrix (prebiotics), was developed (Swanson et al., 2020). Some studies have also found that the effects of probiotics on the host are not directly associated with the active microorganisms but instead are indirectly mediated by the metabolites or bacterial components of certain probiotics (Klemashevich et al., 2014; Salminen et al., 2021), such as SCFAs, which are plant polysaccharide products that are broken down by the gut microbiota (Fang et al., 2022). A study found a synergistic effect between SCFA-producing bacteria and inulin which can improve neurological deficit and behavioral outcomes post-stroke (Lee et al., 2020).

Probiotics and prebiotics are the most extensively studied biotherapeutic strategies to maintain and improve brain function *via* the MGBA (Dinan et al., 2013; Cryan et al., 2019; Martínez-Guardado et al., 2022). Probiotics and prebiotics are strong candidates for treating and preventing IS as they can reshape the gut microbiota, inhibit oxidative stress, and maintain the regular pathways related to microbial metabolism and brain functions. However, most findings and inferences in this field are based on animal studies, and only a few probiotics and prebiotics have been studied (Sarkar et al., 2016) in different combinations for their commercial availability or other physiological beneficial effects, but no study has investigated their specific properties related to the modulation of the MGBA. Therefore, future studies should focus on the mechanisms and targeted effects to improve the brain function of specific probiotic strains and prebiotics.

## Fecal microbiota transplantation (FMT) in IS

Fecal microbiota transplantation is the most efficient intervention to reconstruct the gut microbiota and might be an effective therapeutic strategy for IS. A study found that FMT attenuated cerebral ischemic injury and improved neurological deficit in obese rats, which was probably mediated by the lowering of oxidative stress and apoptosis in the brain (Xie T. et al., 2022). FMT also ameliorated and/or protected transient MCAO mice from transient cerebral ischemic injury (Benakis et al., 2016). *Lactobacillus helveticus* and *Lactobacillus brevis* are the most affected microbiota in ischemia and reperfusion brain injury. Restoration of the *L. helveticus* and *L. brevis* colonies had strong neuroprotective effects. It significantly alleviated the accumulation of branched-chain amino acids (BCAAs), which aggravated microglia-induced neuroinflammation through the AKT/STAT3/NF-kB signaling pathway in the development of IS (Shen et al., 2023). Additionally, as an aged biome can increase the systemic proinflammatory cytokine levels (Spychala et al., 2018), which in turn contributes to the pathogenesis of IS, replenishing the gut microbiome with fresh microorganisms can reverse age-related poor stroke recovery through host immunologic, microbial, and metabolomic modulation.

As a key player in the MGBA, SCFAs can protect against neurodegenerative diseases by regulating the release of hormones and neurotransmitters mediated by G-protein-coupled receptors to further regulate inflammation and the mood of the patient (Fang et al., 2022). Among the known SCFAs, butyric acid showed the highest negative correlation with IS. A recent study reported that administering butyrate decreased exacerbated cerebral infarction in IS associated with type 2 diabetes. The mechanisms related to this effect might include improvements in the functions of the gut barrier and the blood-brain barrier and a decrease in the serum levels of lipopolysaccharides (LPSs), LPS-binding protein (LBP), and proinflammatory cytokines (Wang H. et al., 2022). Interfering with the gut microbiota by transplanting fecal bacteria rich in SCFAs and supplementing with butyric acid could thus be an effective strategy for treating IS (Chen et al., 2019b). In a study, the researchers performed direct enrichment of selective SCFA-producing bacteria, which included *Bifidobacterium longum*, *Clostridium symbiosum*, *Faecalibacterium prausnitzii*, and *Lactobacillus fermentum*. The results showed that these SCFA-producing bacteria alleviated post-stroke neurological deficits and inflammation and increased the concentrations of SCFAs in the gut, brain, and plasma of aged mice after a stroke (Lee et al., 2020). These findings confirmed the effects of a more targeted and refined microbiome therapy.

These studies showed the beneficial effects of FMT on patients with neurological disorders. However, almost all studies were conducted on animal models. Additionally, one study conducted with an animal model for stroke also recorded an increase in mortality after FMT (Vendrik et al., 2020). As the beneficial effects of FMT are not clear, whether positive findings from animal studies can be verified in treating human diseases needs to be ascertained. Large double-blinded randomized controlled trials need to be conducted to further explain the impact of FMT in IS. In recent years, many novel therapeutic strategies targeting specific bacteria have been developed, such as phage therapeutics or multi-phage cocktail therapy, cytokine modulators, and gene therapy. These techniques are more applicable than FMT.

## Traditional Chinese medicine (TCM) in IS

Besides strategies directly modulating the intestinal microbiota, drugs that influence the intestinal microbiota might be more convenient in clinical practice. TCM emphasizes the holistic concept, which is consistent with the modern view of the MGBA in stroke. In China, since the Han Dynasty period, TCM practices have been passed down and evolved over thousands of years, and many classic and effective medicines have been developed for treating IS (Sun et al., 2015). Recent studies have shown that many TCM formulae and monomers exert therapeutic effects by modulating the intestinal microbiota and improving the secretion of gastrointestinal hormones (Zhai et al., 2023).

Traditional Chinese medicine can be used to effectively modulate intestinal homeostasis based on the concept of “homology of medicine and food” and the typical hepatoenteric characteristics of the pharmacokinetic profiles. Terpenoids, glycosides, flavonoids, steroids, polyphenols, and polysaccharides, among other bioactive substances found in TCM, can play distinct roles in multiple gut microbial metabolic pathways (Li X. et al., 2021). These active ingredients in the gut can reshape the structure of the intestinal microflora by increasing beneficial bacteria and decreasing harmful bacteria, thereby facilitating metabolic processes that reduce oxidative stress and inflammation after a stroke (Wang Y. X. et al., 2021).

Here, we briefly summarized the pharmacological effects of natural botanical active ingredients, TCM monomers, and compounds in the pathological state of IS based on the intestinal microbiota and their metabolites, as shown in Table 1. The orally administered TCM primarily interacts with the intestinal microbiota in three ways in IS patients. (1) TCM modulates gut microbiota composition; (2) TCM regulates intestinal metabolites; (3) Intestinal microbiota transforms the components of TCM and improves their metabolism, absorption, and synergism. Specifically, TCM can change the composition and structure of the gut microbiota and affect the production of gut microbiota-associated metabolites. Thus, it exerts anti-inflammatory, anti-oxidative, and immune regulatory effects, which can improve the outcome of IS. Additionally, the intestinal microbiota exerts strong effects on the metabolism of TCM through oxidation, reduction, hydrolysis, and hydroxylation reactions, which are important for improving the absorption of TCM and exerting pharmacological effects (Chen et al., 2016). These findings provide new information that might help elucidate the mechanisms through which TCM affects IS.

**TABLE 1:** The summary of pharmacological effects of herbal ingredients and natural products in IS based on the intestinal microbiota.

Natural products and botanical herbal components	Effects in IS based on intestinal microbiota
**Active ingredients of herbs**	
*Anthraquinones* (Guo Y. et al., 2021)	*Lactobacillus, Bifidobacterium*↑ *Escherichia coli, Enterococcus*↓
**Saponins**	
Astragaloside IV (Yin et al., 2020)	*Clostridium, Blautia, Bifidobacterium,* *Holdemanella, Megamonas* ↑
Ginsenosides (Chen H. et al., 2020)	*Lactobacillus helveticus*↑
Panax notoginseng saponins (Li et al., 2018)	*Bifidobacterium longum*↑
**Herb pair**	
*Chuanxiong-Pueraria* (Chen et al., 2019a)	*Ruminococcaceae_UCG_004,* *Ruminococcaceae_UCG_005,* *Ruminococcaceae_NK4A214_group,* *Lachnospiraceae_NK4B4_group,* *Akkermansia, Alloprevotella,* *Oscillospira, Megasphaera*↑
**TCM prescription**	
Angong Niuhuang Pill (Zhang H. et al., 2022)	*the family Prevotellaceae, the genus* *Alloprevotella, the phylum* *Bacteroidota*↓ *the family Lachnospiraceae, the* *genera Lachnoclostridium, the phylum* *Firmicutes, Enterorhabdus,* *Colidextribacter, Roseburia*, *Lachnospiraceae_UCG-006*↑ prostaglandin I2 and uridine↑
Buyang Huanwu decoction (Liu W. et al., 2022)	*Lactobacilli, Bifidobacteria*↑ *Escherichia coli, Actinobacterium*↓
Dihuang Yinzi (Wang X. et al., 2022)	*Firmicutes, Bacteroidetes,* *Proteobacteria*↑
Huangqi-Honghua (Wang K. et al., 2022)	*Ruminococcaceae, Bacteroides,* *Phascolarctobacterium,* *Desulfovibrionaceae*↓ *Blautia, Lachnospiraceae, Oscillibacter,* *Bifidobacterium*↑ bile acid receptor FXR activated
Huazhuo Jiedu Huoxue Tongluo Prescription (Ni et al., 2022)	*Firmicutes, Bacteroidetes, Lactobacillus,* *Prevotella*↑ *Enterobacteriaceae, Clostridium,* *Enterococcus*↓
Tanhuo decoction (Guo Q. et al., 2021)	*Anaerostipes, Bifidobacterium, Blautia,* *Coprococcus, Gemmiger, Ruminococcus,* *Streptococcus*↑ *Lachnospira, Odoribacter, Eubacterium,* *Phascolarctobacterium*↓
Tong-Qiao-Huo-Xue Decoction (Zhang F. et al., 2020)	*Bacteroidetes, Isobacillus,* *Bifidobacteria*↑ intestinal barrier repaired
Xinglou Chengqi Decoction (Gao et al., 2021)	*Verrucomicrobia, Akkermansia*↑ *Paraprevotella, Roseburia, Streptophyta,* *Enterococcu, Bacteroidetes*↓ short chain fatty acids (SCFAs)↑
Zhilong Huoxue Tongyu capsule (Wang R. et al., 2022)	*Proteobacteria, Prevotella*↑ *Firmicutes, Bacteroidota, Lactobacillus*↓

The benefits of TCM for treating IS based on gut microbiota may be associated with reshaping the gut microenvironment, weakening of bacterial flora translocation, and an increase in probiotics to reduce cerebrovascular damage (Zhang H. Y. et al., 2021). To develop more effective TCM for treating IS, novel gut microbiota sequencing technologies must be used to investigate the gut microbiota for more accurately and precisely assessing the regulatory impact of TCM, as well as to establish more standardized and unified stable IS animal models for determining TCM impact. Furthermore, in various IS models, including rodents and large mammals, the long-term protective effects of TCM on the brain and survival rate and the mechanism of regulating intestinal flora must be determined. Also, the current pharmacokinetics, pharmacodynamics, and toxicological characteristics of TCM require more attention.

Acupuncture treatment at different acupoints, such as Quchi and Zusanli (Ke et al., 2022), is an efficient therapy for IS. It is extensively practiced in China and has also been accepted in other countries and regions in recent years. The mechanism of action of acupuncture might be associated with its effects on intestinal microecology and plasma metabolism. It might influence *Turicibacter*, isoflavones, phytoestrogen metabolites (Xian et al., 2022), and IPA levels (Li et al., 2022). Additionally, the combination of acupuncture and TCM might have synergistic effects, which might further enhance the recovery of IS when administered together.

## Intestinal epithelial stem cell transplants (gut-derived stem cells) in IS

Several studies have shown an association between a leaky gut and alterations in gut microbiota in patients with IS (Huang and Xia, 2021; Zhang W. et al., 2022). The leaky gut hypothesis suggests that the increase in gut permeability might cause inflammatory cytokines and toxic gut metabolites to pass through the compromised intestinal epithelial barrier. The resultant endotoxemia and bacterial translocation can aggravate gut hemorrhage, gut dysmotility, intestinal paralysis, bowel incontinence, and even gut-origin sepsis, along with neurological impairment and a series of secondary injuries after IS (Larochelle et al., 2022; Zhang W. et al., 2022). Therefore, the intestinal epithelium needs to be repaired for the recovery of the patient after a stroke. Stem cell therapy and organoid techniques are novel strategies for gut remediation (Shaker and Rubin, 2012). Mani et al. (2023) showed that the gut is a critical therapeutic target for stroke. They engrafted organoids containing intestinal epithelial stem cells (IESCs) from young rats into older model rats that suffered a stroke. They found that the transplanted IESCs incorporated into the gut restored gut dysbiosis caused by the stroke and decreased intestinal permeability, which reduced the circulating levels of endotoxin LPS and the inflammatory cytokine IL-17A. They also discovered that IESC transplantation improved stroke-induced acute (4 day) sensory-motor disability as well as chronic (30 day) cognitive-affective function. The findings emphasized the importance of early intervention in the acute stage of stroke and transplantation of IESCs from young people. However, no clinical studies on the efficacy of gut-derived stem cells in the treatment of IS have been reported in the literature to date. In the future, it will be critical to investigate donor selection, the mechanisms underlying cell engraftment, and regimens to maximize transplant efficiency. Therefore, further investigation is needed to optimize the transplantation time, dose, and route to apply gut stem cell therapy in the clinic.

## Summary

**FIGURE 1:**
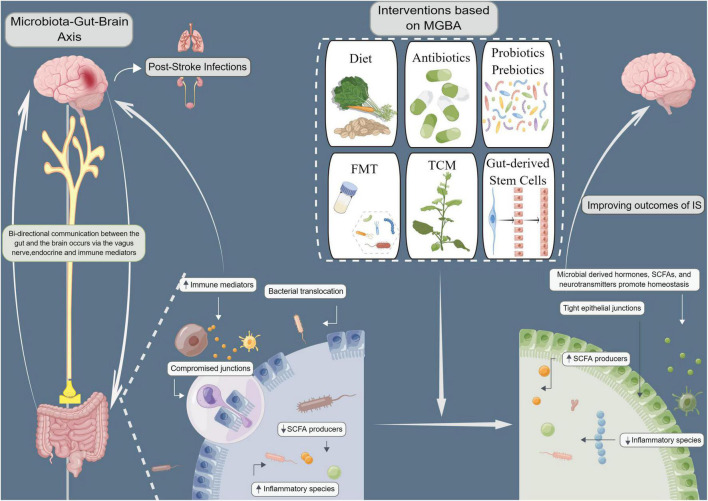
Improving ischemic stroke outcomes (IS) with microbiota-gut-brain axis (MGBA)-based interventions. Microbiological interventions, including dietary interventions, antibiotics, probiotics and prebiotics, fecal microbiota transplantation (FMT), traditional Chinese medicine (TCM), and intestinal stem cell transplantation can improve MGBA by altering microbial communities. The gut microbiome is known to be highly involved in the biosynthesis and release of various hormones, neurotransmitters, and numerous active metabolites and agents that may directly or indirectly regulate MGBA *via* neurobiological networks, immunological processes, and/or microbial metabolic signaling pathways, thereby affecting brain function and systemic inflammation. Modulation of gut microbiota composition and microbiota-derived metabolites may prevent infectious complications and improve neurological outcomes in IS patients by increasing short-chain fatty acids (SCFAs) and neurochemicals, decreasing gut permeability, reducing bacterial translocation, and alleviating immunosuppression.

The gut shows an early response to stroke, and changes in the gut occur simultaneously with stroke-induced hyperpermeability of the BBB. After the concept of MGBA was proposed, several studies showed the high clinical application value of the approaches targeting intestinal microbiota in the treatment of IS. The gut microbiota can influence the metabolic status of the body besides exerting strong effects on blood pressure, blood glucose, and atherosclerosis, all of which are risk factors for IS (Wang J. et al., 2022). A detailed study of the physiological functions of the gut microbiota and gut microbiota disorders associated with the central nervous system might provide new ideas for preventing and treating IS. Additionally, several studies have also investigated the development of the dietary intervention, antibiotics, probiotics and prebiotics, FMT, TCM, as well as gut-derived stem cells for the microbiome-based treatment of IS (Figure 1). However, intestinal microbiota-targeted treatment of IS needs further improvement. Large-sample multicenter studies with long-term follow-up need to be conducted to verify the benefits. Identifying specific species of pathogenic bacteria, optimizing targeted regimens, and combining therapies can greatly contribute to the advancements in treating IS.

## Author contributions

QL and XL contributed to the conception and design of the study. JW wrote the first draft of the manuscript. JW, QL, and XL wrote sections of the manuscript. All authors contributed to the manuscript revision, read, and approved the submitted version.
